# Intravenous Immunoglobulin Therapy Restores the Quantity and Phenotype of Circulating Dendritic Cells and CD4^+^ T Cells in Children With Acute Kawasaki Disease

**DOI:** 10.3389/fimmu.2022.802690

**Published:** 2022-02-10

**Authors:** Nana Wang, Zhongyue Chen, Fan Zhang, Qianwen Zhang, Ling Sun, Haitao Lv, Bo Wang, Jie Shen, Xufang Zhou, Feiyan Chen, Binwei Zhang, Lijun Meng, Huiting Zhou, ZhenJiang Bai, Jie Huang

**Affiliations:** ^1^Department of Cardiology, Children’s Hospital of Soochow University, Suzhou, China; ^2^Department of Hematology, Children’s Hospital of Soochow University, Suzhou, China; ^3^Pediatric Research Institute of Soochow University, Suzhou, China; ^4^Department of Pediatric Intensive Care Unit, Children Hospital of Soochow University, Suzhou, China

**Keywords:** Kawasaki disease, intravenous immunoglobulin, dendritic cells, CD4^+^ T cells, immune disorders

## Abstract

**Background:**

Intravenous immunoglobulin (IVIG) showed its therapeutic efficacy on Kawasaki disease (KD). However, the mechanisms by which it reduces systemic inflammation are not completely understood. Dendritic cells (DCs) and T cells play critical roles in the pathogenic processes of immune disorders. Assessing the quantity of DC subsets and T cells and identifying functional molecules present on these cells, which provide information about KD, in the peripheral blood may provide new insights into the mechanisms of immunoglobulin therapy.

**Methods:**

In total, 54 patients with KD and 27 age-matched healthy controls (HCs) were included in this study. The number, percentage, and phenotype of DC subsets and CD4^+^ T cells in peripheral blood were analyzed through flow cytometry.

**Results:**

Patients with KD exhibited fewer peripheral DC subsets and CD4^+^ T cells than HCs. Human leucocyte antigen-DR (HLA-DR) expression was reduced on CD1c^+^ myeloid DCs (CD1c^+^ mDCs), whereas that on plasmacytoid DCs (pDCs) did not change significantly. Both pDCs and CD1c^+^ mDCs displayed significantly reduced expression of co-stimulatory molecules, including CD40, CD86. pDCs and CD1c^+^ mDCs presented an immature or tolerant phenotype in acute stages of KD. Number of circulating pDC and CD1c^+^ mDC significantly inversely correlated with plasma interleukin-6 (IL-6) levels in KD patients pre-IVIG treatment. No significant differences were found concerning the DC subsets and CD4^+^ T cells in patients with KD with and without coronary artery lesions. Importantly, these altered quantity and phenotypes on DC subsets and CD4^+^ T cells were restored to a great extent post-IVIG treatment. T helper (Th) subsets including Th1 and Th2 among CD4^+^ T cells did not show alteration pre- and post-IVIG treatment, although the Th1-related cytokine IFN-γ level in plasma increased dramatically in patients with KD pre-IVIG treatment.

**Conclusions:**

pDCs and CD1c^+^ mDCs presented an immature or tolerant phenotype in acute stages of KD, IVIG treatment restored the quantity and functional molecules of DCs and CD4^+^ T cells to distinct levels *in vivo*, indicating the involvement of DCs and CD4^+^ T cells in the inflammation in KD. The findings provide insights into the immunomodulatory actions of IVIG in KD.

## Introduction

Kawasaki disease (KD) is a febrile systemic vasculitis that predominately affects children less than 5 years of age and is the leading cause of acquired cardiac disorders in children (1). Increasing evidence supports that immune-mediated inflammation plays an essential role in the pathophysiology of KD. However, to date, the mechanisms involved in the etiology of KD have not been completely elucidated. It has been thought to be triggered by different antigens that causes a series of aberrant innate and adaptive immune responses (2). Dendritic cells (DCs) are professional antigen presenting cells playing a key role in inducing the activation of naive T cells and bridging innate and adaptive immunity (3–7). DCs recognize the presence of pathogens through pattern recognition receptors, including TLRs, and further migrate from the periphery to the lymph nodes to present pathogen-derived antigens to T cells. Studies have indicated that DCs and T cells could play a key role in KD pathogenesis because mature and activated DCs and T cells expressing the HLA-DR antigen have been reported to infiltrate the coronary artery and skin lesions in patients with KD (8, 9) and in the LCCWE-induced coronary artery lesion mouse model (10). The previous experiments clearly demonstrate that KD leads to a decline in DC numbers (11); however, the maturation status of the surviving DCs remain unclear. Altered activation and effector subsets (Th1 and Th2) have been reported but no consistent conclusion has been drawn yet.

Intravenous immunoglobulin (IVIG) treatment remains the most effective therapy currently for KD. Although the underlying mechanisms are not fully elucidated, it is well-accepted that IVIG cures patients by down-regulating inflammatory responses, which further protects the vascular system and myocardium from immune-mediated damage. Diverse mechanisms have been suggested to explain the anti-inflammatory activity of IVIG, including the neutralization of microbial toxins or cytokines (12), suppression of T- and B- cell activation (13), promotion of apoptosis of peripheral blood lymphocytes (14), regulation of Th17/Treg cell balance (15), and reduction in cytokine release (16, 17). However, only a few studies have investigated the action of IVIG on DCs and CD4^+^ T cells in KD. Systemic studies on DCs and T cells are warranted to gain insights into the role of these immune cells in the KD pathogenesis and IVIG treatment.

In this study, we determined the frequency, number, phenotype of peripheral pDC and CD1c^+^ mDC, expression of the inhibitory receptors on CD4^+^ T cells and helper T cell subsets in patients suffering from KD and the changes of these parameters over the course of IVIG treatment.

## Materials and Methods

### Participants

A total of 54 with KD admitted to the Department of Cardiology, Children’s Hospital of Soochow University, from June 2020 to June 2021 participated in this study. All the participants conformed to the diagnostic criteria revised by AHA Kawasaki Disease Guideline (18). All patients received intravenous immunoglobulin (IVIG,2 g/kg) in addition to oral aspirin (30-50 mg/kg/day) as an initial treatment. Patients who fulfilled at least one of the following criteria were excluded: 1) having received corticosteroids and immunosuppressive drugs, 2) having received their initial IVIG infusion in other hospitals, 3) refused IVIG infusion, and 4) IVIG resistance, 5) recurrent KD. Echocardiography was performed in all the patients before the IVIG treatment. Measurements of the diameter of the left main coronary artery (LCA), the anterior descending branch (LAD), the circumflex artery (CX), and the right coronary artery (RCA)were normalized based on the body surface area and expressed as a Z score (standard deviation units from the mean). A Z score ≥ 2 was to indicate coronary artery lesion (19). In total, 27 age-matched healthy subjects were enrolled as controls who had received routine regular physical examinations were enrolled as healthy controls (HCs). Clinical parameters such as the white blood cells (WBC), neutrophil count, lymphocyte count, C reactive protein (CRP) level, and Prealbumin were recorded from both patients and healthy controls. The parents of all the study participants were informed about the study, and they provided informed consent.

### Flow Cytometry Analysis

Peripheral blood (2 mL) was collected from both healthy controls (HCs) and patients at two time points: before IVIG treatment (pre-IVIG) and 3 days after completing initial IVIG treatment (post-IVIG). Peripheral blood leukocytes were isolated from EDTA-blood through red blood cell lysis within 4 h. Briefly, red blood cells (RBCs) were lysed using human erythrocyte lysing solution (BioLegend), and samples were washed twice with phosphate-buffered saline (PBS) without Ca2^+^ and Mg2^+^. After the samples were washed, peripheral blood leukocytes were resuspended in PBS containing 2.5% fetal bovine serum (FBS) and incubated at 4°C for 30 min in the dark with the following fluorochrome-conjugated monoclonal antibodies including surface CD3-FITC (SK7), CD14-FITC (HCD14), CD15-FITC (HI98), CD16-FITC (3G8), CD19-FITC (HIB19), CD20-FITC (2H7), CD56-FITC (MEM-188), HLA-DR-PE/CY7 (L243), CD123-APC(6H6), CD1c-PE(L161), CD86-PE/CY7(IT2.2), CD40-BV421(5C3), CD4-BV421 (RPA-T4), Tim3-APC (F38-2F2), TIGIT-PE (A15153G), and PD-1-BV510 (NAT105).All the samples were washed and analyzed using the Attune NxTflow cytometer (LifeTechnology). Approximately 3 × 10^6^ and 1 × 10^6^ of peripheral blood leukocytes were used to analyze DCs and CD4^+^ T cells by flow cytometry, respectively. All antibodies were purchased from BioLegend (San Diego, CA, USA) or BD Biosciences (San Diego, CA, USA). Staining was performed as previously described (20). Total DCs (Pan-DCs) were characterised as negative for lineage markers (CD3, CD14, CD15, CD16, CD19, CD20, and CD56) and positive for HLA-DR. The myeloid DCs and plasmacytoid DCs subsets were characterised as CD1c^+^ and CD123^+^, respectively. Dead cells were excluded through 7-aminoactinomycin D (7-AAD; BioLegend) viability staining. The absolute number of CD1c^+^ mDCs and pDCs was calculated from the peripheral blood leukocytes count multiplied by the proportion of each subset within peripheral blood leukocytes per milliliter, as determined by flow cytometric analysis.

### Intracellular Cytokine Determination of CD4^+^ T Cells

For the detection of intracellular cytokines by flow cytometry, peripheral blood leukocytes were cultured in 10% fetal calf serum in RPMI-1640 and incubated for 5 h at 37°C under 5% CO_2_ in the presence of 5 ng/mL phorbol myristate acetate (PMA), 0.5 μg/mL ionomycin, and Golgiplug (containing monensin, 1/1000 final concentration). Cells were washed and stained with anti-CD4 at 4°C for 30 min in the dark. After washed twice, cells were fixed with lysing buffer (BD Biosciences), permeabilized with permeabilization solution (BD Biosciences), and were then incubated with antibodies against IFN-γ and IL-4 (BD Biosciences) for intracellular staining, according to the manufacturer’s instructions. Th1 and Th2 cells were identified as CD4^+^IFN-γ^+^ and CD4^+^IL-4^+^, respectively. Dead cells were excluded using a fixable live/dead dye (Invitrogen). The data were analyzed using FlowJo v10.4 software (FlowJo, OR, USA).

### Plasma Cytokine Measurement

The Human Th1/Th2/Th17 Kit (BD Biosciences) was used to measure plasma cytokine levels. IL-2, IL-4, IL-6, IFN-γ, TNF-α, IL-17A, and IL-10 levels were assessed. Data were analyzed using CBA software. The individual cytokine concentrations were indicated by their fluorescence intensities. The concentrations of all the cytokines were reported in picogram per milliliter.

### Statistical Analysis

Statistical analysis was performed using IBM SPSS Statistics 26.0. All data are expressed as median (interquartile range). For comparison between patients with KD and HCs, Mann-Whitney U-test was used. The Wilcoxon signed-rank test for paired samples was used for comparing the patients before and after IVIG treatment. The significance of difference in sex distribution in patients and controls was analyzed using the chi-square test. Correlations were analyzed using the Spearman correlation test. A *P* value of < 0.05 was considered significant.

## Results

### Baseline Characteristics

A total of 54 patients with KD and 27 healthy controls were recruited based on our inclusion and exclusion criteria. Their characteristics are shown in **Table 1**. No significant differences in age and sex were observed between the groups. The WBC and neutrophil count and prealbumin and CRP levels were significantly higher in patients with KD before IVIG treatment than in HCs, whereas no significant difference was observed in the absolute lymphocyte count. After IVIG treatment, WBC and neutrophil counts and the CRP level decreased rapidly to an almost normal level, whereas the prealbumin level remained lower. According to echocardiography parameters, 52 patients with KD were divided into two groups: KD without coronary artery lesion (CALs; KD-NCAL) group (n = 40) and KD with CAL (KD-CAL) group (n = 14) (**Table 2**). No significant differences were observed in terms of WBC, neutrophil, and lymphocyte counts; neutrophil to lymphocyte ratio (NLR); and CRP and prealbumin levels between the KD-CAL and KD-NCAL groups.

**Table 1:** Characteristics of the study population.

Parameters	Kawasaki disease		Healthy controls
	Pre-IVIG	Post-IVIG	
Number	54	54	27
Age, months	35.5 (18.75-56.25)	35.5 (18.75-56.25)	45 (36-56)
Sex, male/female	29/25	29/25	16/11
Fever duration before diagnosis	5 (4.0-6.25)	5 (4.0-6.25)	–
WBC, 10^9^/L	13.67 (10.59-15.95)^†,‡^	7.54 (5.41-10.47)	7.38 (6.16-8.33)
Neutrophil, 10^9^/L	8.21 (6.09-11.36)^†,‡^	2.59 (1.6-3.9)	2.58 (2.23-3.35)
Lymphocytes,10^9^/L	3.21 (2.52-4.12)	3.38 (2.66-4.88)	3.68 (2.89-4.08)
CRP, mg/L	49.04 (34.42-85.57)^†,‡^	5.9 (3.12-11.86)^†^	0.16 (0.11-0.42)
NLR	2.52 (1.79-3.88)^†,‡^	0.78 (0.52-1.02)	0.77 (0.63-1.09)
Prealbumin, mg/L	75.5 (58.0-87.25)^†,‡^	166 (137.75-200)^†^	207 (190-225)

Baseline characteristics of the participants. Data shown are median (quartile spacing) or the number of cases. -, this data was not detected; CRP, C-reactive protein; NLR, neutrophil to lymphocyte ratio; WBC, white blood cell counts.

^†^P < 0.05 vs. the healthy controls. ^‡^P < 0.05 vs. the post-IVIG treatment

**Table 2:** Characteristics of patients with KD classified according to coronary artery lesions or not.

Parameters	Kawasaki disease		*P* value
	NCAL	CAL	
Number	40	14	
Age, months	33.5 (20.0-50.0)	45.4 (14.5-66.75)	0.4125
Sex, male/female	22/18	7/7	0.7468
Fever duration before diagnosis	6 (4-6.75)	4.5 (4-6.25)	0.2937
WBC, 10^9^/L	13.67 (10.14-15.97)	13.96 (11.43-15.77)	0.5737
Neutrophil, 10^9^/L	8.16 (6.09-11.95)	8.37(6.09-11.10)	0.8979
Lymphocytes,10^9^/L	3.17 (2.53-4.11)	3.32 (2.19-4.30)	0.6427
CRP, mg/L	45.72 (29.14-79.61)	67.24 (38.95-105.28)	0.2690
NLR	2.52 (1.82-3.78)	2.36 (1.61-4.30)	0.7596
Prealbumin, mg/L	76 (59.5-88)	71.5 (52.25-80.25)	0.2645

Baseline characteristics of the participants. Data shown are median (quartile spacing) or the number of cases. CAL, coronary artery lesion; CRP, C-reactive protein; NCAL, without coronary artery lesion; NLR, neutrophil to lymphocyte ratio; WBC, white blood cell counts.

### IVIG Treatment Restored the Distribution of DC Subsets

**Figure 1:**
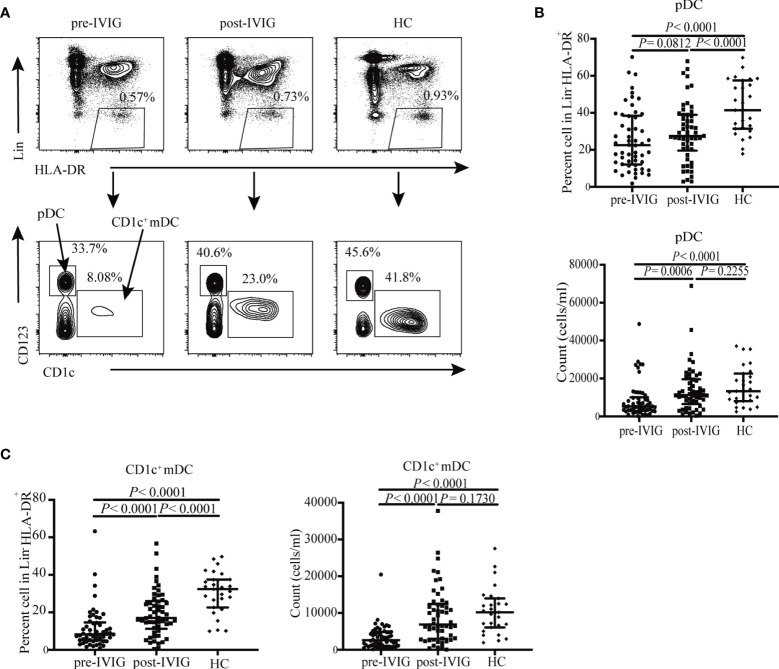
DC subsets distribution in HCs (n = 27) and KD patients pre- and post-IVIG treatment (n = 54). **(A)** Identification of circulating DC subsets in blood using flow cytometry, Pan-DCs were gated as Lin^-^ HLA-DR^+^, pDCs and CD1c^+^ mDCs were defined as Lin^−^ HLA-DR^+^ CD123^+^ and Lin^−^ HLA-DR^+^ CD1c^+^ cells, respectively. Representative profiles of the circulating DCs subsets are shown. **(B)** Plots show percentage and number of circulating pDCs in patients with KD and controls. **(C)** Plots show percentage and number of circulating CD1c^+^ mDCs in patients with KD and HCs. Horizontal bars represent median values, and error bars represent the interquartile range.

To identify DC subsets, the gating strategy of total DCs and their subsets was shown in **Figure 1A**. Quantitative flow cytometric analysis revealed that the frequency and absolute number of pDCs and CD1c^+^ mDCs among pan-DCs was significantly decreased in patients with KD pre-IVIG compared with those in healthy controls (*P* < 0.0001, **Figure 1B**; *P* < 0.0001, **Figure 1B**; *P* < 0.0001, **Figure 1C**; *P* < 0.0001, **Figure 1C**; respectively). This means that the distribution of DC subsets was different from that of HCs, with reduced frequency and number of pDCs and CD1c^+^ mDCs. Importantly, we found significantly recovered frequency and absolute number of pDCs and CD1c^+^ mDCs in patients post IVIG therapy (*P* = 0.0812, **Figure 1B**; *P* = 0.0006, **Figure 1B**; *P* < 0.0001, **Figure 1C**; *P* < 0.0001, **Figure 1C**; respectively). Although the percentage of both DC subsets in patients with KD did not recover to their levels observed in HCs (*P* < 0.0001, **Figure 1B**; *P* < 0.0001, **Figure 1C**; respectively), the absolute number of both DC subsets was same as that found in HCs (*P* = 0.2255, **Figure 1B**; *P* = 0.1730, **Figure 1C**; respectively).

### IVIG Induced Phenotypic Changes of Circulating DCs in KD Patients

Next, we compared the expressions of the antigen presenting molecule HLA-DR and co-stimulatory molecules (CD40 and CD86) in the two DC subsets in the peripheral circulation of patients with KD and HCs. These molecules are critical for DCs to elicit adaptive immune responses.

**Figure 2:**
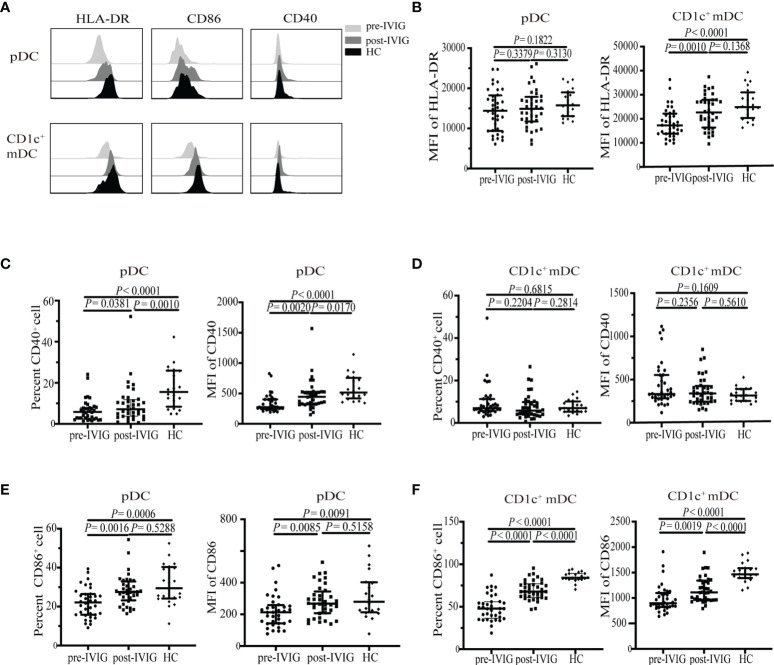
Expression of HLA-DR, CD86 and CD40 on circulating DC subsets from healthy control (HC) (n = 20) and KD patients pre-and post-IVIG treatment (n = 36). **(A)** Representative histograms of HLA-DR, CD86, CD40 in DC subsets from a healthy control and KD patients pre-and post-IVIG treatment. **(B)** HLA-DR MFI on pDCs and CD1c^+^ mDCs in KD patients and HCs. **(C, D)** Percentage and MFI of CD40^+^ on pDCs and CD1c^+^ mDCs in patients with KD and HCs. **(E, F)** Percentage and MFI of CD86^+^ on pDCs and CD1c^+^ mDCs in patients with KD and HCs. Horizontal bars represent median values, and error bars represent the interquartile range. MFI, mean fluorescent intensity.

Although no significant significance was observed between pre-and post-IVIG treatment in patients with KD and HCs in terms of pDCs, the MFI of the antigen presenting molecule HLA-DR exhibited a decreasing trend (*P* = 0.1822, **Figure 2B**; *P* = 0.3130, **Figure 2B**; respectively). CD40 percentage on pDCs in patients pre-IVIG treatment and MFI considerably decreased (*P* < 0.0001, **Figure 2C**; *P* < 0.0001, **Figure 2C**; respectively). IVIG treatment significantly elevated the percentage and MFI of CD40 expression on pDCs; however, they were not at normal levels as observed in HCs (*P* = 0.0010, **Figure 2C**; *P* = 0.0170, **Figure 2C**; respectively). Similarly, we found that the percentage and MFI of CD86 expression on pDCs were significantly lower in patients with KD pre-IVIG treatment than those in HCs (*P* = 0.0006, **Figure 2E**; *P* = 0.0091, **Figure 2E**; respectively). The percentage and MFI of CD86 in pDC was increased in patients after IVIG treatment, with no difference between patients post-IVIG treatment and HCs (*P* = 0.5288, **Figure 2E**; *P* = 0.5158, **Figure 2E**; respectively).

For CD1c^+^ mDCs, we observed significantly decreased MFI of HLA-DR in patients with KD pre-IVIG treatment compared with that in HC, with no difference between post treatment and HCs (*P* < 0.0001, **Figure 2B**; *P* = 0.1368, **Figure 2B**; respectively). Differing from pDCs, drastic change of co-stimulatory molecule on CD1c^+^ mDCs in patients was CD86 instead of CD40. A drastic decrease in the MFI and of CD86 percentage was observed on CD1c^+^ mDCs in the patients before treatment (*P* < 0.0001, **Figure 2F**; *P* < 0.0001, **Figure 2F**; respectively). IVIG treatment significantly elevated the percentage and MFI of CD86 expression on CD1c^+^ mDCs in patients with KD; however, these parameters were significantly lower than those in HCs (*P* < 0.0001, **Figure 2F**; *P* < 0.0001, **Figure 2F**; respectively). We did not observe obvious changes in terms of the percentage and MFI of CD40 on CD1c^+^ mDCs between patients with KD and HCs (*P* = 0.6815, **Figure 2D**; *P* = 0.1609, **Figure 2D**; respectively).

Collectively, data presented in **Figures 1** and **2** indicated that the quantity of DCs and functional molecules on DCs were impaired in patients pre-IVIG treatment. IVIG treatment restored the quantity of DCs and functional molecules on DCs to distinct levels, indicating a possible role of DCs in the recovery of patients after IVIG treatment.

### TIM-3^+^ CD4^+^ T Cells Are Reduced Post-IVIG Treatment

**Figure 3:**
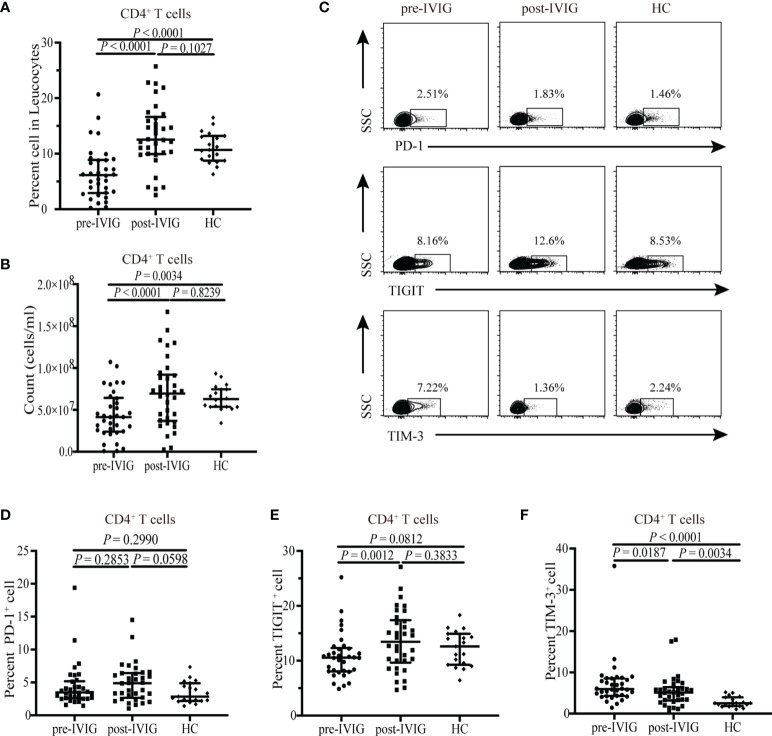
Flow cytometry detection of the expression of PD-1, TIGIT, and TIM-3 on CD4^+^ T cells from healthy control (HC) (n = 19) and KD patients pre- and post-IVIG treatment (n = 34). Graphs show **(A, B)** Percentage and number of CD4^+^ T cells among peripheral blood leucocytes in KD patients and HCs. **(C)** Representative dot plots of PD-1, TIGIT, and TIM-3 are shown on gated CD4^+^ T cells. **(D)** Expression of PD-1 on CD4^+^ T cells in patients with KD and HCs. **(E)** Expression of TIGIT on CD4^+^ T cells in patients with KD and HCs. **(F)** Expression of TIM-3 expression on CD4^+^ T cells in patients with KD and HCs. Horizontal bars represent median values, and error bars represent the interquartile range. MFI, mean fluorescent intensity.

Considering the impaired quantity and functional molecules on DCs in patients with acute KD patients, we further assessed CD4^+^ T cells because DCs are the critical regulators of CD4^+^ T cells. The percentage and absolute number of CD4^+^ T cells were decreased in the peripheral blood of patients pre-IVIG treatment compared with those in HCs (*P* < 0.0001, **Figure 3A**; *P* = 0.0034, **Figure 3B**; respectively). Inhibitory receptors such as programmed cell death 1 (PD-1), T cell immunoglobulin and ITIM domain (TIGIT), and T cell immunoglobulin and mucin domain 3 (TIM-3) are important molecules controlling T cell effector responses (21). Inhibitory receptors have been involved in the pathophysiology of various human diseases, including autoimmune diseases (21, 22), sepsis (23), and cancer (24). However, the expression of these inhibitory receptors on CD4^+^ T cells has not been reported previously in KD. To determine whether these inhibitory receptors are involved in the pathogenesis of KD, we used flow cytometry to assess the expressions of PD-1, TIGIT, and TIM-3 on peripheral CD4^+^ T cells. Representative flow cytometric analyses are shown in **Figure 3C**, patients with KD were found to exhibit a significantly higher expression of TIM-3 on CD4^+^ T cells pre-IVIG treatment than HCs, whereas no changes in PD-1 or TIGIT were observed between them (*P* < 0.0001, **Figure 3F**; *P* = 0.2990, **Figure 3D**; *P* = 0.0812, **Figure 3E**; respectively). IVIG treatment significantly reduced percentage TIM-3 expression on CD4^+^ T cells but not to a normal level as seen in HCs (*P* = 0.0034, **Figure 3F**). The percentage of CD4^+^ T cells expressing the TIGIT was increased post IVIG treatment (*P* = 0.0012, **Figure 3E**).

### Unaltered Th1/Th2 Polarization of CD4^+^ T Cells in KD Patients

**Figure 4:**
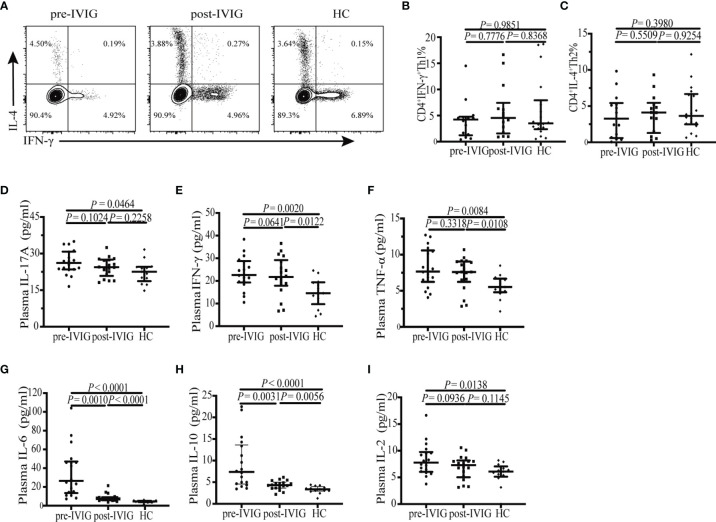
Flow cytometry analysis of the intracellular cytokine and plasma cytokine levels. **(A)** The gating strategy for determining CD4^+^ IFN-γ^+^ Th1 and CD4^+^ IL-4^+^ Th2 cells is shown. **(B)** Percentage of circulating CD4^+^ IFN-γ^+^ Th1 cells in patients with KD (n = 14) and HCs (n = 18). **(C)** Percentage of circulating CD4^+^ IL-4^+^ Th2 cells in patients with KD (n = 14) and HCs (n = 18). **(D–I)** Plasma levels of the cytokines IL-17A, IFN-γ, TNF-α, IL-6, IL-10, IL-2 were detected in patients with KD (n = 19) and HCs (n = 13) using cytometric bead array. Horizontal bars represent median values, and error bars represent the interquartile range.

Although many studies have focused on T helper cytokines, most of them have determined the cytokines in plasma instead of CD4^+^ T cells. To understand the biology of CD4^+^ T cells, frequencies of different CD4^+^ T-cell subsets were analyzed based on cytokine patterns after *in vitro* stimulation of the T-cell receptor (TCR) signal. Production of intracellular IFN-γ and IL-4, which are the representative factors of Th1 and Th2, respectively, in CD4^+^ T cells, was analyzed in the peripheral blood of patients with KD and HCs. The gating strategy for determining Th1 and Th2 cells is shown in **Figure 4A**. Neither the onset of KD nor IVIG treatment altered the percentages of Th1 and Th2 in CD4^+^ T cells (*P* = 0.9851, **Figure 4B**; *P* = 0.7776, **Figure 4B**; *P* = 0.3980, **Figure 4C**; *P* = 0.5509, **Figure 4C**; respectively).

We further measured the level of a panel of Th subset–related cytokines in the plasma of patients with KD and HCs. Of these, the plasma level of IL-4 was very low or undetectable in either study population (date not shown). IL-17A, IFN-γ, TNF-α, IL-6, IL-10, and IL-2 levels were higher in patients with KD pre-IVIG than in HCs (*P* = 0.0464, **Figure 4D**; *P* = 0.0020, **Figure 4E**; *P* = 0.0084, **Figure 4F**; *P* < 0.0001, **Figure 4G**; *P* < 0.0001, **Figure 4H**; *P* = 0.0138, **Figure 4I**; respectively). These results are consistent with those previously reported (15, 25). After IVIG treatment, plasma levels of IL-17A and IL-2 returned to normal (*P* = 0.2258, **Figure 4D**; *P* = 0.1145, **Figure 2I**; respectively), the levels of other cytokines including IFN-γ, TNF-α, IL-6, and IL-10 did not decrease markedly but slightly deviated from normal levels (*P* = 0.0122, **Figure 4E**; *P* = 0.0108, **Figure 2F**; *P* < 0.0001, **Figure 4G**; *P* = 0.0056, **Figure 4H**; respectively).

### Increased Level of IL-6 in Plasma Correlated With Decreased Number of pDC and CD1c^+^ mDC in KD Patients Pre-IVIG Treatment

**Figure 5:**
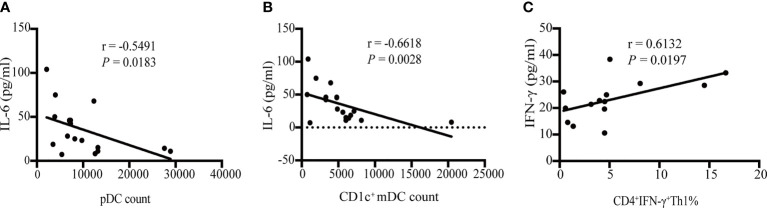
Spearman correlation between cytokines and immunocytes. **(A)** Correlation analysis of IL-6 level and pDC count in KD patients pre-IVIG treatment (n = 18). **(B)** Correlation analysis of IL-6 level and CD1c^+^ mDC count in KD patients pre-IVIG treatment (n = 18). **(C)** Correlation analysis of IFN-γ level and the percentage of circulating CD4^+^ IFN-γ^+^ Th1 in KD patients pre-IVIG treatment (n = 14).

In order to analyze the correlation between cytokines and immunocytes, we performed the Spearman correlation analysis. In our study, only plasma IL-6 level significantly inversely correlated with circulating pDC count (r = -0.5491, *P* = 0.0183) (**Figure 5A**) and CD1c^+^ mDC count (r = -0.6618, *P* = 0.0028) (**Figure 5B**), while no correlations were found between IL-6 level and CD4^+^ T cell count in KD patients pre-IVIG treatment (**Supplementary Table 1**). There were no significant correlations between the number of pDC, CD1c^+^ mDC and CD4^+^ T cell and cytokines (IL-17A, IFN-γ, TNF-α, IL-10 and IL-2) (**Supplementary Table 1**). After IVIG treatment, neither the number of pDC, CD1c^+^ mDC, nor CD4^+^ T cell correlated significantly with the cytokines (**Supplementary Table 2**). We next analyzed the correlation between cytokines and the percentage of circulating Th subsets. Plasma levels of IFN-γ positively correlated with the percentage of circulating Th1 in KD patients pre-IVIG treatment (r = 0.6132, *P* = 0.0197) (**Figure 5C**), while no correlations were found between the percentage of Th1 and other cytokines (IL-17A, TNF-α, IL-10, IL-6 and IL-2). There were no significant correlations between the percentage of circulating Th2 and cytokines (**Supplementary Table 3**). Following treatment, neither the percentage of Th1, nor Th2 correlated significantly with the cytokines (**Supplementary Table 4**).

### Numbers and Phenotypic Properties on DC Subsets and CD4^+^ T Cells in Patients With CAL and Without CAL Pre-IVIG Treatment

To investigate the correlation among DC subsets, CD4^+^ T cells, and CAL, we performed a subgroup analysis comparing patients with and without CAL (KD-CAL and KD-NCAL groups). The number and proportion of circulating pDCs and CD1c^+^ mDCs were not significantly different in KD-CAL and KD-NCAL groups (**Table 3**). The expressions of HLA-DR, CD86, and CD40 on pDCs and CD1c^+^ mDCs did not differ significantly between KD-CAL and KD-NCAL groups (**Table 4**). Furthermore, no difference was observed in the number, proportion, and TIM-3 receptor expression of CD4^+^ T cells between the two groups (**Table 5**).

**Table 3:** Percentage and number of DC subsets between KD patients with and without CAL.

Parameters	Kawasaki disease		*P* value
	NCAL (n = 40)	CAL (n = 14)	
pDCs%	18.45 (11.82-32.28)	31.15 (14.24-41.48)	0.1671
pDCs (/ml)	4964 (2979-7212)	10425 (4024-24450)	0.0725
CD1c^+^ mDCs%	7.84 (4.72-14.93)	8.44 (5.69-15.28)	0.8668
CD1c^+^ mDCs (/ml)	2440 (900-4846)	3146 (1688-4937)	0.6498

Data shown are median (quartile spacing) or the number of cases. CAL, coronary artery lesion; NCAL, without coronary artery lesion.

**Table 4:** HLA-DR, CD86 and CD40 expression on DC subsets between KD patients with and without CAL.

Parameters	Kawasaki disease		*P* value
	NCAL (n = 29)	CAL (n = 13)	
MFI of HLA-DR on pDCs	11826 (8605-15739)	16625 (13268-18988)	0.0586
MFI of HLA-DR on CD1c^+^ mDCs	16360 (13777-21717)	18561 (14376-22444)	0.5586
CD86^+^ expression on pDCs%	22.6 (15.8-27.2)	20.5 (15.25-28.0)	0.7543
CD86^+^ expression on CD1c^+^ mDCs%	47.8 (38.56-56.2)	47.7 (34.4-60.15)	0.6534
MFI of CD86 on pDCs	224 (142-294)	204 (149-242)	0.6147
MFI of CD86 on CD1c^+^ mDCs	915 (850-1145)	967 (738-1102)	0.3914
CD40^+^ expression on pDCs%	4.20 (2.05-7.61)	6.07 (3.41-11.65)	0.0866
CD40^+^ expression on CD1c^+^ mDCs%	8.29 (6.21-17.65)	6.45 (5.21-12.1)	0.1310
MFI of CD40 on pDCs	274 (239-404)	341 (274-430)	0.0997
MFI of CD40 on CD1c^+^ mDCs	430 (318-1007)	304 (265-577)	0.0643

Data shown are median (quartile spacing) or the number of cases. CAL, coronary artery lesion; NCAL, without coronary artery lesion.

**Table 5:** CD4^+^ T cells and TIM3^+^ CD4^+^ T cells between KD patients with and without CAL.

Parameters	Kawasaki disease		*P* value
	NCAL (n = 25)	CAL (n = 9)	
CD4^+^ T cells %	6.47 (4.68-9.71)	3.83 (2.30-7.29)	0.0859
CD4^+^ T cells (/ml)	42034115 (24620739-62163389)	41448706 (24919683-74148861)	0.9842
TIM-3^+^ expression on CD4^+^ T cells %	5.58 (4.20-7.81)	6.93 (4.92-8.73)	0.2717

Data shown are median (quartile spacing) or the number of cases. CAL, coronary artery lesion; NCAL, without coronary artery lesion.

## Discussion

**Figure 6:**
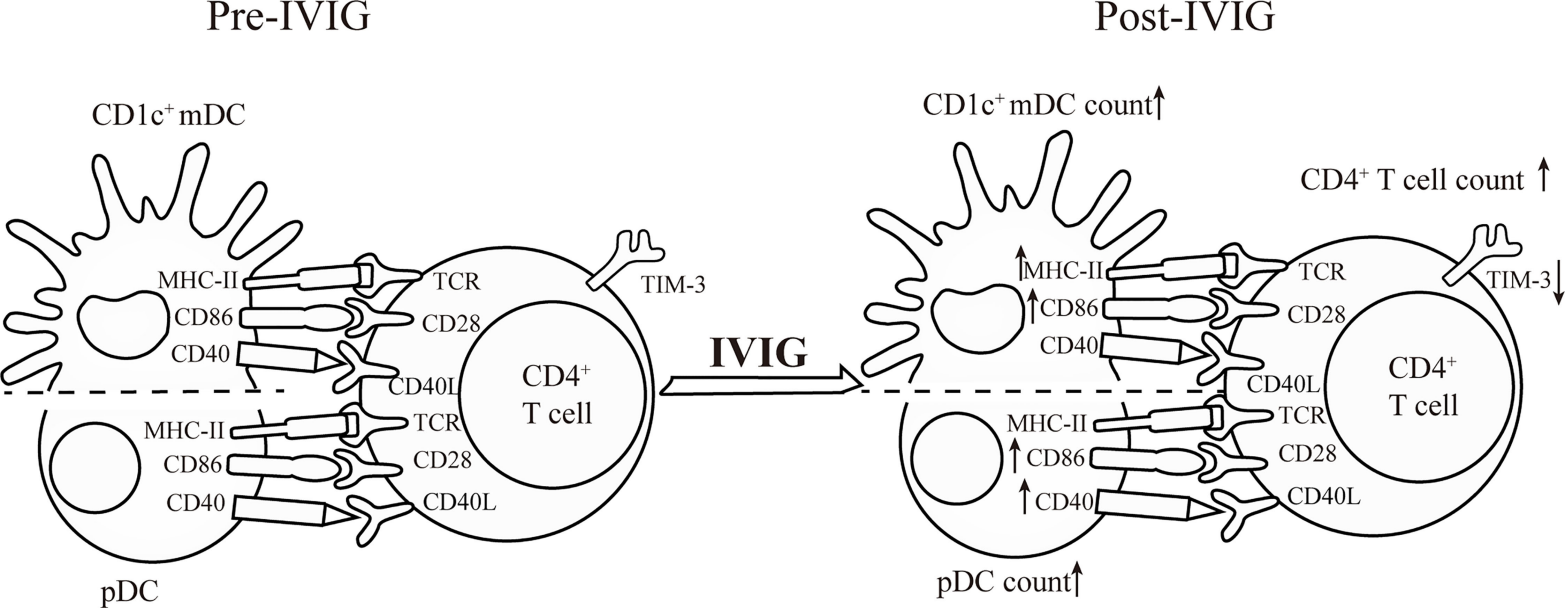
Cartoon chart shows the effects of IVIG on the quantity and functional molecules of DC subsets and CD4^+^ T cells in KD patients.

In this study, we reported that the frequency and number of pDCs and CD1c^+^ mDCs and the expression of the antigen presenting molecule HLA-DR and co-stimulatory molecules, CD86 and/or CD40, on DCs decrease in acute stages of KD. IVIG treatment restored the quantity of DCs and functional molecules on DCs to distinct levels. In addition, our data indicated that the frequency and number of CD4^+^ T cells decreased in peripheral blood leukocytes in patients pre-IVIG treatment and were restored to a normal level post-IVIG treatment. The expression of the inhibitory receptor TIM-3 on peripheral CD4^+^ T cells increased in patients pre-IVIG treatment and decreased post- IVIG treatment but not to a normal level (**Figure 6**). Although the levels of characteristic inflammatory cytokines including IL-2, IL-6, IFN-γ, TNF-α, IL-17A, and IL-10 in patients with acute KD increased significantly compared with those in HCs, which is consistent with previous reports, we did not observe any changes in Th subsets when gated on peripheral CD4^+^ T cells. No significant differences were observed concerning the quantity and phenotype of DC subsets and CD4^+^ T cells in patients with KD with and without CALs. Moreover, we also found circulating numbers of pDC and CD1c^+^ mDC significantly inversely correlated with plasma IL-6 levels, and plasma IFN-γ levels were positively associated with the percentage of circulating Th1 in KD patients pre-IVIG treatment.

For DC subsets, contradictory results have been reported in humans regarding numerical abnormalities of peripheral blood DC subtypes in KD (11, 26). Suda et al. reported a decreased number of circulating mDCs but not of pDCs (11). Burns et al. reported an increase in circulating mDCs but not in pDCs in the acute phase of KD (26). Such contradictory results may be related to the different markers used for DC classification because DCs were identified through Lin^-^ (CD3, CD14, CD15, CD16, CD19, CD20, CD56) and HLA-DR staining, the makers of DC staining in our study are consistent with those of previous report (19). However, Suda et al. (11) identified DCs as Lin^-^ (CD3, CD14, CD16, CD19, CD20, CD56) and HLA-DR. Burns et al. (26) relied upon CD11b and CD11c markers to identify DCs. Reduction in the number of DCs may be because of alterations in DC viability, impaired differentiation with progenitor cells, or altered tissue distribution under inflammation, although the precise mechanism remains to be further validated. Studies have suggested that DCs migrate from the periphery to the site of coronary artery injury, given that mature and activated DCs infiltrate and accumulate around the coronary arteries. In conjunction with alterations in the percentage and absolute number of DC subsets, markers expressed on the surface of DCs that reflect their function are also significantly altered in KD. Expression of HLA-DR, a professional antigen presenting molecule, decreased significantly on both pDCs and CD1c^+^ mDCs in KD. We observed down-regulated expressions of CD40 and CD86 on pDCs in patients with KD compared with those in HCs. Similarly, CD1c^+^ mDCs also exhibited significantly lower levels of CD86; however, the expression of CD40 did not differ compared with that in HCs. Studies have established that mature DCs display a phenotype characterised by high surface expression of antigen presenting molecules and co-stimulatory molecules such as CD86 and CD40 (27, 28). Immature DCs are known to have a lower potential to activate T cells (29). Our results indicated that the circulating pDCs and CD1c^+^ mDCs in patients with KD might reflect a tolerant or less mature phenotype in this condition. Importantly, studies on humans and animal models have indicated the presence of mature and activated DCs in CALs (8), which is probably due to an increased efflux of more mature DCs into the affected coronary artery and/or an increased influx of less mature DCs from the bone marrow. However, the assumption requires further validation.

The patients in our study exhibited a profoundly decreased percentage and number of CD4^+^ T cells, consistent with a previous report by Lee et al. (30). Numerous immunological studies on peripheral blood lymphocytes have been conducted, however, the role of T cells and the functional state of Th1 and Th2 cells remains controversial. Matsubara et al. reported a decrease in the number of Th1 type CD3^+^ T cell in the peripheral blood of patients with acute stage of KD and suggested the presence of a Th1/Th2 imbalance, particularly Th2 dominance (31). Lee et al. reported that both Th1 and Th2 cells may be activated simultaneously during the acute stage of KD (25), whereas Kimura et al. suggested that the production of Th1 and Th2 cytokines is suppressed at the level of transcriptional regulation in KD (32). Interestingly, we did not observe any change in Th subsets among CD4^+^ T cells between patients with KD and HCs. Lee et al. (25) determined the cytokines in plasma instead of CD4^+^ T cells. Kimura et al. (32) analyzed mRNA levels of T-bet and GATA-3, along with those of IFN-γ and IL-4, in peripheral blood mononuclear cells but not in CD4^+^ T cells. Th1 (IFN-γ-producing CD4^+^ T cells) and Th2 (IL-4-producing CD4^+^ T cells) cells were identified by intracellular cytokine staining in our study. These experimental differences, as well as other methodological differences may account for the discrepancy. The function of T cells is regulated by inhibitory receptors such as PD-1, TIGIT, and TIM-3 (21). In this regard, none of the studies have reported the expression of PD-1, TIGIT, and TIM-3 expression in human CD4^+^ T cells. Augmented TIM-3 expression was observed on CD4^+^ T cells, whereas no changes in PD-1 and TIGIT expressions were observed in patients with KD prior to treatment with those in HCs. Given that TIM-3 is a negative regulatory molecule on CD4^+^ T cells, patients with KD might exhibit CD4^+^ T cells in a more suppressed state than HCs.

However, no significant differences were found concerning the quantity and phenotype of DC subsets and CD4^+^ T cells in patients with and without CAL in the acute phase of KD. Studies have reported the associations of CALs with several clinical variables, particularly CRP and NLR. In our clinical data, patients with CAL exhibited no significant elevation in CRP and NLR. The reason for these discrepant results is unclear; however, it may be related to various factors including different genetic backgrounds (33), pathogen species (34), and epigenetic effects (35).

Overall, these results strongly implicated that pDCs, CD1c^+^ mDCs and CD4^+^ T cells are in a suppressed state in the acute phase of KD. Our results are not consistent with the findings of Burns et al. (26), who reported an increased number of mDCs expressing CD86 in the acute KD phase. This difference might be because of the inclusion of CD14-positive cells, since monocytes have CD14 on their cell surface, whereas mDCs generally lack CD14. It remains uncertain whether peripheral blood T cells are activated in acute KD phases. The increased expression of TIM-3 on CD4^+^ T cells as observed in patients with KD may be associated with the suppression of the immune response. Our results are consistent with those of a study by Kuijpers et al. (36), who reported that a dysregulated TcR/CD3-dependent T cell unresponsiveness in acute KD. Additionally, Matsubara et al. (37) suggested that T cells in the peripheral blood of patients with KD were not activated because of the low expression levels of intracellular CTLA-4. Ikeda et al. (38) performed the microarray analysis of peripheral blood mononuclear cells and reported that the expressions of genes involved in antigen processing and presentation, the TCR signalling pathway, and the B-cell receptor signalling pathway were downregulated in acute phase KD. Thus, the decline in the number of pDCs and CD1c^+^ mDCs in patients with KD and their immature phenotype and the increase in TIM-3 expression on CD4^+^ T cells suggested a deficiency in the defensive system of patients with KD and may account for a high infection rate in these patients.

The mechanisms of action of IVIG in KD have been studied extensively; however, the role of DCs and CD4^+^ T cells in the resolution of inflammation in response to IVIG treatment has rarely been investigated. A study demonstrated the stimulation of normal donor tolerogenic mDCs *in vitro*, with Fc-induced IL-10 secretion, which influenced the differentiation of T cells to regulatory T cells; this finding indicated that the protective effects of IVIG treatment were, at least, partly mediated by Fc (26). In this study, the number of circulating pDCs and CD1c^+^ mDCs increased to the normal levels after IVIG treatment. Moreover, IVIG treatment restored the expression of HLA-DR, CD86, and CD40 on DC subsets to different degrees. In addition, the CD4^+^ T cells were affected by IVIG treatment. The percentage of CD4^+^ T cells expressing the inhibitory receptor TIM-3 was decreased after IVIG treatment. A previous study reported that TIM-3 expression on Treg has been associated with increased suppressive Treg activity (39). IVIG can both modulate Treg cell function and increase Treg cell expansion (15). The reason for this inconsistency may be that the TIM-3 expression is not limited to Treg cells, and it has been shown to be expressed on Th1 cells and CD8^+^ T cytotoxic type 1 cells (21, 40). Nonetheless, the expression of TIM-3, which is considered an important immuno-inhibitory receptor, in any cell subgroup is correlated with the induction of the immunosuppressive function (41). According to our study, however, the total level of TIM-3 expression on CD4^+^ T cells was significantly decreased (although to a small extent) post-IVIG therapy; and according to the data on the plasma cytokines, the level of immunosuppressive molecule IL-10 was significantly decreased post-IVIG therapy. Thus, we speculate that the restoration of the cell number and phenotype of CD4^+^ T cells is involved in the recovery of the immune system of the patients post- IVIG therapy to a certain degree. To the best of our knowledge, this study is the first to describe the variation in inhibitory receptor expression on CD4^+^ T cells in KD. These results demonstrated that IVIG induced multiple phenotypic and functional changes in DC subsets and CD4^+^ T cells, mainly by promoting the resolution of inflammation, in patients with KD. The specific mechanisms leading to these effects should be further investigated.

In this study, we found that elevation of plasma IL-6 was associated with decreased number of pDC and CD1c^+^ mDC in patients with KD pre-IVIG treatment. A previous study by Dawicki et al. demonstrated that IL-6 act to selectively promote the accumulation of DC subsets into an inflamed lymph node in mouse model in response to bacterial peptidoglycan (42). Alternatively, Lin et al. showed that IL-6 plays an important role in the apoptosis of cDC1 in mouse model of pancreatic cancer (43). We speculate that IL-6 elevation in KD may be associated with increased recruitment of DC subsets from blood to the affected tissues or increased DCs apoptosis, which awaits further verification. Additionally, there was a significant positive correlation between Th1 and IFN-γ. IFN-γ is one of the major functional cytokines produced by Th1, of note, IFN-γ is also important for Th1 maintenance and production (44, 45). This is in accordance with our findings that peripheral Th1 and plasma IFN-γ were positively correlated.

Our study has some limitations. First, this preliminary study included a relatively small cohort of patients. Large-scale, multi-centre prospective studies are needed to confirm our findings. Second, this study involved short-term IVIG treatment; therefore, additional studies are required to elucidate the effects of IVIG on DC subsets and T cells throughout the entire inflammatory phase. Third, a further analysis of the function of these circulating DCs in patients with KD was hampered by a small number of DCs in the peripheral circulation. Hence, to confirm the functional characteristics of DCs and CD4^+^ T cells, further *in vitro* and animal studies are needed. Fourth, we could not analyse the relationship between IVIG-nonresponsive and DC subsets and CD4^+^ T cells because our study did not incorporate this study group. These issues will be addressed in our subsequent studies.

Collectively, our data indicated that IVIG could restore the percentage, number, and expression of effector molecules in DC subsets. Similarly, the frequencies and absolute numbers of abnormally reduced CD4^+^ T cells increased. Moreover, the expression of TIM-3 on CD4^+^ T decreased after IVIG treatment, suggesting that IVIG played a role in inhibiting TIM-3 expression, which might be one of the mechanisms of action of IVIG in KD treatment. Changes in DC subsets and CD4^+^ T cells contributed to the restoration of the immune balance, which can be used as an indicator of clinical monitoring of the immune status in patients with KD. This study offers us an insight into KD pathogenesis and provides a new perspective for understanding the mechanisms of action of IVIG.

## Data Availability Statement

The original contributions presented in the study are included in the article/**Supplementary Material**. Further inquiries can be directed to the corresponding author.

## Ethics Statement

The experimental protocols were established following the Declaration of Helsinki and approved by the Ethics Committee of Children’s Hospital of Soochow University. Written informed consent to participate in this study was provided by the participants’ legal guardian/next of kin.

## Author Contributions

JH and LM designed and supervised the study and revised the manuscript. FZ, QZ, XZ, FC, and BZ collected clinical information and samples. LM and NW wrote the manuscript. NW and ZC carried out the experiments, and analyzed, and interpreted the data. LM, ZB, HZ, LS, HL, BW, and JS interpreted the data and discussed the results, which are vital for the formation of conception. All authors participated in the manuscript review. The authors read and approved the final manuscript.

## Funding

This work is supported by the National Natural Science Foundation of China (31670853), the Natural Science Foundation of Jiangsu Province (BK20190053), the Medical Science Program of Jiangsu Province (H2019002), the Talent Engineering Project of Jiangsu Province (BRA2018393), the Science and Technology Project of Jiangsu Province (BE2019672), the Suzhou Clinical Medicine Expert Project (GSWS2019015), the Science and Technology Program of Suzhou (SYS2018067), and the Postgraduate Research & Practice Innovation Program of Jiangsu Province (KYCX20_2725).

## Conflict of Interest

The authors declare that the research was conducted in the absence of any commercial or financial relationships that could be construed as a potential conflict of interest.

## Publisher’s Note

All claims expressed in this article are solely those of the authors and do not necessarily represent those of their affiliated organizations, or those of the publisher, the editors and the reviewers. Any product that may be evaluated in this article, or claim that may be made by its manufacturer, is not guaranteed or endorsed by the publisher.

## Abbreviations

CBA, Cytometric bead array; DCs, Dendritic cells; HCs, Healthy controls; FBS, fetal bovine serum HLA-DR, Human leucocyte antigen-DR; IVIG, intravenous immunoglobulin; IL, Interleukin; IFN-γ, Interferon-γ; KD, Kawasaki disease; mDCs, myeloid dendritic cells; pDCs, plasmacytoid dendritic cells; MFI, Mean fluorescence intensity; NLR, neutrophil to lymphocyte ratio; PD-1, Programmed cell death 1; PMA, phorbol myristate acetate; TIM-3, T-cell immunoglobulin and mucin domain 3; Th, T helper; TIGIT, T-cell immunoglobulin and ITIM domain; TNF-α, Tumor necrosis factor-α; 7-AAD, 7-aminoactinomycin D.
