# Supplementary material for: Intravenous Immunoglobulin Therapy Restores the Quantity and Phenotype of Circulating Dendritic Cells and CD4+ T Cells in Children With Acute Kawasaki Disease
Source: Front Immunol. 2022 Feb 10;13:802690. doi: 10.3389/fimmu.2022.802690 (PMC8866170; doi:10.3389/fimmu.2022.802690)
Supplement: Supplementary file 1 [file DataSheet_1.docx]

**Supplementary Table 1** Correlation between cytokines and pDC count, CD1c^+^ mDC count and CD4^+^ T cell count in KD patients pre-IVIG treatment (n=18).

| Variable | pDC count | | CD1c^+^ mDC count | | CD4^+^ T cell count | |
| --- | --- | --- | --- | --- | --- | --- |
|  | r | *P*-value | r | *P*-value | r | *P*-value |
| IL-17A | 0.3344 | 0.1750 | 0.3644 | 0.1371 | -0.1574 | 0.5329 |
| IFN-γ | -0.1246 | 0.6223 | -0.2125 | 0.3972 | -0.1546 | 0.5402 |
| [TNF-α](http://www.baidu.com/baidu.php?url=Ks00000EAMrnlPLIyofT73NTz3RavFsFuJGWGNhfkYCO2MSlj_kkFR2kLfYvWZ3TCnQioC87jKb5pxq4VEIf6DVUsGQX3gziGltSnsZ0BceEbisSP4YFJznQ9pv5j_SoPVfhEiojRk_OZFLCCIxIKne_nRg_FB0Ndlr43AXUeRLlaSlxAcbrHZQs-1_m7RCO7T4SBoWfOvqnvDC1V7cdxGQkwiAq.7D_NR2Ar5Od663rj6tV2UCpblRqArunTxFKnh7vhqtHnNj5lpagkwsRcVXIhWWJu1YcVTXEFMqPKQ3_S-vaP55EZPakxIMVyFWuvyU8PhOuztXyeR_nYQZHYe70.U1Yk0ZDqIA4hFHFDG_D0TA-W5H00TZPGuv3qPWRdmHP9PjDYmWK9uyuWn1m4mHuWuH9hnyc4nyDkPHc0IjdYUhm-nbw9UZKCmfKGUHYznWR0u1dEugK1nfKdpHdBmy-bIykV0ZKGujYkrfKWpyfqPHb0UgfqnH0krNtknjDLg1csPH7xnW0vn-t1PW0k0AVG5H00TMfqPWn30AFG5HDdr7tznjwxPH010AdW5HD3PWcvrHTvrH9xnHbknjc4PWb1P7tznjRkg1cLnjbzPj6vrHnLg100TgKGujYs0Z7Wpyfqn0KzuLw9u1Ys0A7B5HKxn0K-ThTqn6KsTjYkPjbLnW0krHbs0A4vTjYsQW0snj0snj0s0AdYTjYs0AwbUL0qn0KzpWYk0Aw-IWdsmsKhIjYs0ZKC5H00ULnqn0KBI1YknfK8IjYs0ZPl5fK9TdqGuAnqTZnVuyPJ0A-bm1dribGH0ZKCIZbq0Zw9ThI-IjYvndtsg1DdnsKYIgnqnHDdrHc3P10YnHD1rjfLPWRsnHm0ThNkIjYkPWfsn10sPjTdPW0d0ZPGujdbuWw-rAfsn10snj03m1w90AP1UHdaPjKjwW03nRD1nRR4nYcL0A7W5HD0TA3qn0KkUgfqn0KkUgnqn0KlIjYz0AdWgvuzUvYqn7tsg1Kxn7ts0Aw9UMNBuNqsUA78pyw15HKxn7tsg1Kxn0Ksmgwxuhk9u1Ys0AwWpyfqn0K-IA-b5iYk0A71TAPW5H00IgKGUhPW5H00Tydh5H00uhPdIjYs0A-1mvsqn0K9uAu_myTqnfK_uhnqn0KbmvPb5H0zwRfkrHDknYDYP1F7f1FafW0kPW9Anb7af16vnjfzwbRsPHckrDR3HdFiHb4af-w7ifKYTh7buHYvn10znjc0mhwGujYYnHfdnY77rH-7wDmLPRDLfRwKwHFjnWf3wH04wHNar0KBIjYs0Aq9IZTqn0KEIjYz0AqzTZfqninsc1DWPan4n1bzPj61P16WnansQW0snj0snansc1fWnanVc108nj0snj0sc1D8nj0snH0s0Z91IZRqPWRknjnsP0KkgLmqna3dnNtsQW0sg108njKxna3snNtsQW0zg108rHNxna3zn-ts0AF1gLKzUvwGujYs0ZFEpyu_myTqn0KWIWY0pgPxmLK95H00mL0qn0K-TLfqn0KWThnqnWbvPjb&us=newvui&xst=TjYkPjbLnW0krHbs0ynqnjF7wjD4nHD1fHfLnbNjnbFanjDvrDmzfRFjrjmsPjFAwH0dnWD3wH95R-FrHbFaNDNF0ycqPjDYPHPKwHb4wRwAP1NKPY7DfRRzf1cYrDRsrRRdfW6KT1YkPW04njbdn1RvnWmvPH6kPHDkP7tznWNxn07L5gw8uBd9UZKCmf7k5gw8uBvhsf7d5HmdnH01njfKIjYkPWfsn10sPjTd0ydk5H0an0cV0yPC5yuWgLKW0ykd5H0Kmv3qrjfsPHnsrH9xndqbusDkPjfdnjD1nHD4&word=&ck=1425.2.0.0.0.214.140.0&shh=www.baidu.com&sht=84053098_3_dg&wd=) | 0.2042 | 0.4163 | 0.2353 | 0.3473 | -0.2952 | 0.2343 |
| IL-6 | -0.5491 | 0.0183 | -0.6618 | 0.0028 | -0.2854 | 0.2509 |
| IL-10 | 0.1153 | 0.6487 | -0.0708 | 0.7800 | 0.0657 | 0.7957 |
| IL-2 | 0.1406 | 0.5780 | 0.3535 | 0.1501 | 0.0021 | 0.9935 |

**Supplementary Table 2** Correlation between cytokines and pDC count, CD1c^+^ mDC count and CD4^+^ T cell count in KD patients post-IVIG treatment (n=18).

| Variable | pDC count | | CD1c^+^ mDC count | | CD4^+^ T cell count | |
| --- | --- | --- | --- | --- | --- | --- |
|  | r | *P*-value | r | *P*-value | r | *P*-value |
| IL-17A | 0.3175 | 0.1992 | 0.2420 | 0.3333 | 0.0465 | 0.8545 |
| IFN-γ | -0.0403 | 0.8738 | 0.0527 | 0.8354 | -0.3814 | 0.1184 |
| [TNF-α](http://www.baidu.com/baidu.php?url=Ks00000EAMrnlPLIyofT73NTz3RavFsFuJGWGNhfkYCO2MSlj_kkFR2kLfYvWZ3TCnQioC87jKb5pxq4VEIf6DVUsGQX3gziGltSnsZ0BceEbisSP4YFJznQ9pv5j_SoPVfhEiojRk_OZFLCCIxIKne_nRg_FB0Ndlr43AXUeRLlaSlxAcbrHZQs-1_m7RCO7T4SBoWfOvqnvDC1V7cdxGQkwiAq.7D_NR2Ar5Od663rj6tV2UCpblRqArunTxFKnh7vhqtHnNj5lpagkwsRcVXIhWWJu1YcVTXEFMqPKQ3_S-vaP55EZPakxIMVyFWuvyU8PhOuztXyeR_nYQZHYe70.U1Yk0ZDqIA4hFHFDG_D0TA-W5H00TZPGuv3qPWRdmHP9PjDYmWK9uyuWn1m4mHuWuH9hnyc4nyDkPHc0IjdYUhm-nbw9UZKCmfKGUHYznWR0u1dEugK1nfKdpHdBmy-bIykV0ZKGujYkrfKWpyfqPHb0UgfqnH0krNtknjDLg1csPH7xnW0vn-t1PW0k0AVG5H00TMfqPWn30AFG5HDdr7tznjwxPH010AdW5HD3PWcvrHTvrH9xnHbknjc4PWb1P7tznjRkg1cLnjbzPj6vrHnLg100TgKGujYs0Z7Wpyfqn0KzuLw9u1Ys0A7B5HKxn0K-ThTqn6KsTjYkPjbLnW0krHbs0A4vTjYsQW0snj0snj0s0AdYTjYs0AwbUL0qn0KzpWYk0Aw-IWdsmsKhIjYs0ZKC5H00ULnqn0KBI1YknfK8IjYs0ZPl5fK9TdqGuAnqTZnVuyPJ0A-bm1dribGH0ZKCIZbq0Zw9ThI-IjYvndtsg1DdnsKYIgnqnHDdrHc3P10YnHD1rjfLPWRsnHm0ThNkIjYkPWfsn10sPjTdPW0d0ZPGujdbuWw-rAfsn10snj03m1w90AP1UHdaPjKjwW03nRD1nRR4nYcL0A7W5HD0TA3qn0KkUgfqn0KkUgnqn0KlIjYz0AdWgvuzUvYqn7tsg1Kxn7ts0Aw9UMNBuNqsUA78pyw15HKxn7tsg1Kxn0Ksmgwxuhk9u1Ys0AwWpyfqn0K-IA-b5iYk0A71TAPW5H00IgKGUhPW5H00Tydh5H00uhPdIjYs0A-1mvsqn0K9uAu_myTqnfK_uhnqn0KbmvPb5H0zwRfkrHDknYDYP1F7f1FafW0kPW9Anb7af16vnjfzwbRsPHckrDR3HdFiHb4af-w7ifKYTh7buHYvn10znjc0mhwGujYYnHfdnY77rH-7wDmLPRDLfRwKwHFjnWf3wH04wHNar0KBIjYs0Aq9IZTqn0KEIjYz0AqzTZfqninsc1DWPan4n1bzPj61P16WnansQW0snj0snansc1fWnanVc108nj0snj0sc1D8nj0snH0s0Z91IZRqPWRknjnsP0KkgLmqna3dnNtsQW0sg108njKxna3snNtsQW0zg108rHNxna3zn-ts0AF1gLKzUvwGujYs0ZFEpyu_myTqn0KWIWY0pgPxmLK95H00mL0qn0K-TLfqn0KWThnqnWbvPjb&us=newvui&xst=TjYkPjbLnW0krHbs0ynqnjF7wjD4nHD1fHfLnbNjnbFanjDvrDmzfRFjrjmsPjFAwH0dnWD3wH95R-FrHbFaNDNF0ycqPjDYPHPKwHb4wRwAP1NKPY7DfRRzf1cYrDRsrRRdfW6KT1YkPW04njbdn1RvnWmvPH6kPHDkP7tznWNxn07L5gw8uBd9UZKCmf7k5gw8uBvhsf7d5HmdnH01njfKIjYkPWfsn10sPjTd0ydk5H0an0cV0yPC5yuWgLKW0ykd5H0Kmv3qrjfsPHnsrH9xndqbusDkPjfdnjD1nHD4&word=&ck=1425.2.0.0.0.214.140.0&shh=www.baidu.com&sht=84053098_3_dg&wd=) | -0.0072 | 0.9772 | -0.2453 | 0.3265 | -0.0062 | 0.9805 |
| IL-6 | 0.3445 | 0.1615 | 0.0217 | 0.9318 | 0.1997 | 0.4269 |
| IL-10 | 0.2316 | 0.3550 | 0.0414 | 0.8705 | 0.3495 | 0.1551 |
| IL-2 | 0.1503 | 0.5516 | 0.0010 | 0.9967 | 0.2644 | 0.2891 |

**Supplementary Table 3** Correlation between cytokines and the percentage of circulating Th subsets in KD patients pre-IVIG treatment (n=14).

| Variable | CD4^+^ IFN-γ^+^ Th1^+^% | | CD4^+^ IL-4^+^ Th2^+^% | |
| --- | --- | --- | --- | --- |
|  | r | *P*-value | r | *P*-value |
| IL-17A | -0.1694 | 0.5626 | -0.3300 | 0.2492 |
| IFN-γ | 0.6132 | 0.0197 | 0.1253 | 0.6696 |
| [TNF-α](http://www.baidu.com/baidu.php?url=Ks00000EAMrnlPLIyofT73NTz3RavFsFuJGWGNhfkYCO2MSlj_kkFR2kLfYvWZ3TCnQioC87jKb5pxq4VEIf6DVUsGQX3gziGltSnsZ0BceEbisSP4YFJznQ9pv5j_SoPVfhEiojRk_OZFLCCIxIKne_nRg_FB0Ndlr43AXUeRLlaSlxAcbrHZQs-1_m7RCO7T4SBoWfOvqnvDC1V7cdxGQkwiAq.7D_NR2Ar5Od663rj6tV2UCpblRqArunTxFKnh7vhqtHnNj5lpagkwsRcVXIhWWJu1YcVTXEFMqPKQ3_S-vaP55EZPakxIMVyFWuvyU8PhOuztXyeR_nYQZHYe70.U1Yk0ZDqIA4hFHFDG_D0TA-W5H00TZPGuv3qPWRdmHP9PjDYmWK9uyuWn1m4mHuWuH9hnyc4nyDkPHc0IjdYUhm-nbw9UZKCmfKGUHYznWR0u1dEugK1nfKdpHdBmy-bIykV0ZKGujYkrfKWpyfqPHb0UgfqnH0krNtknjDLg1csPH7xnW0vn-t1PW0k0AVG5H00TMfqPWn30AFG5HDdr7tznjwxPH010AdW5HD3PWcvrHTvrH9xnHbknjc4PWb1P7tznjRkg1cLnjbzPj6vrHnLg100TgKGujYs0Z7Wpyfqn0KzuLw9u1Ys0A7B5HKxn0K-ThTqn6KsTjYkPjbLnW0krHbs0A4vTjYsQW0snj0snj0s0AdYTjYs0AwbUL0qn0KzpWYk0Aw-IWdsmsKhIjYs0ZKC5H00ULnqn0KBI1YknfK8IjYs0ZPl5fK9TdqGuAnqTZnVuyPJ0A-bm1dribGH0ZKCIZbq0Zw9ThI-IjYvndtsg1DdnsKYIgnqnHDdrHc3P10YnHD1rjfLPWRsnHm0ThNkIjYkPWfsn10sPjTdPW0d0ZPGujdbuWw-rAfsn10snj03m1w90AP1UHdaPjKjwW03nRD1nRR4nYcL0A7W5HD0TA3qn0KkUgfqn0KkUgnqn0KlIjYz0AdWgvuzUvYqn7tsg1Kxn7ts0Aw9UMNBuNqsUA78pyw15HKxn7tsg1Kxn0Ksmgwxuhk9u1Ys0AwWpyfqn0K-IA-b5iYk0A71TAPW5H00IgKGUhPW5H00Tydh5H00uhPdIjYs0A-1mvsqn0K9uAu_myTqnfK_uhnqn0KbmvPb5H0zwRfkrHDknYDYP1F7f1FafW0kPW9Anb7af16vnjfzwbRsPHckrDR3HdFiHb4af-w7ifKYTh7buHYvn10znjc0mhwGujYYnHfdnY77rH-7wDmLPRDLfRwKwHFjnWf3wH04wHNar0KBIjYs0Aq9IZTqn0KEIjYz0AqzTZfqninsc1DWPan4n1bzPj61P16WnansQW0snj0snansc1fWnanVc108nj0snj0sc1D8nj0snH0s0Z91IZRqPWRknjnsP0KkgLmqna3dnNtsQW0sg108njKxna3snNtsQW0zg108rHNxna3zn-ts0AF1gLKzUvwGujYs0ZFEpyu_myTqn0KWIWY0pgPxmLK95H00mL0qn0K-TLfqn0KWThnqnWbvPjb&us=newvui&xst=TjYkPjbLnW0krHbs0ynqnjF7wjD4nHD1fHfLnbNjnbFanjDvrDmzfRFjrjmsPjFAwH0dnWD3wH95R-FrHbFaNDNF0ycqPjDYPHPKwHb4wRwAP1NKPY7DfRRzf1cYrDRsrRRdfW6KT1YkPW04njbdn1RvnWmvPH6kPHDkP7tznWNxn07L5gw8uBd9UZKCmf7k5gw8uBvhsf7d5HmdnH01njfKIjYkPWfsn10sPjTd0ydk5H0an0cV0yPC5yuWgLKW0ykd5H0Kmv3qrjfsPHnsrH9xndqbusDkPjfdnjD1nHD4&word=&ck=1425.2.0.0.0.214.140.0&shh=www.baidu.com&sht=84053098_3_dg&wd=) | 0.1826 | 0.5321 | -0.0308 | 0.9167 |
| IL-6 | -0.1100 | 0.7081 | 0.2222 | 0.4451 |
| IL-10 | -0.3190 | 0.2662 | 0.3894 | 0.1687 |
| IL-2 | -0.0901 | 0.7593 | -0.1429 | 0.6261 |

**Supplementary Table 4** Correlation between cytokines and the percentage of circulating Th subsets in KD patients post-IVIG treatment (n=14).

| Variable | CD4^+^IFN-γ^+^Th1^+^% | | CD4^+^IL-4^+^Th2^+^% | |
| --- | --- | --- | --- | --- |
|  | r | *P*-value | r | *P*-value |
| IL-17A | 0.1144 | 0.6969 | -0.1452 | 0.6204 |
| IFN-γ | -0.1604 | 0.5838 | -0.0549 | 0.8520 |
| [TNF-α](http://www.baidu.com/baidu.php?url=Ks00000EAMrnlPLIyofT73NTz3RavFsFuJGWGNhfkYCO2MSlj_kkFR2kLfYvWZ3TCnQioC87jKb5pxq4VEIf6DVUsGQX3gziGltSnsZ0BceEbisSP4YFJznQ9pv5j_SoPVfhEiojRk_OZFLCCIxIKne_nRg_FB0Ndlr43AXUeRLlaSlxAcbrHZQs-1_m7RCO7T4SBoWfOvqnvDC1V7cdxGQkwiAq.7D_NR2Ar5Od663rj6tV2UCpblRqArunTxFKnh7vhqtHnNj5lpagkwsRcVXIhWWJu1YcVTXEFMqPKQ3_S-vaP55EZPakxIMVyFWuvyU8PhOuztXyeR_nYQZHYe70.U1Yk0ZDqIA4hFHFDG_D0TA-W5H00TZPGuv3qPWRdmHP9PjDYmWK9uyuWn1m4mHuWuH9hnyc4nyDkPHc0IjdYUhm-nbw9UZKCmfKGUHYznWR0u1dEugK1nfKdpHdBmy-bIykV0ZKGujYkrfKWpyfqPHb0UgfqnH0krNtknjDLg1csPH7xnW0vn-t1PW0k0AVG5H00TMfqPWn30AFG5HDdr7tznjwxPH010AdW5HD3PWcvrHTvrH9xnHbknjc4PWb1P7tznjRkg1cLnjbzPj6vrHnLg100TgKGujYs0Z7Wpyfqn0KzuLw9u1Ys0A7B5HKxn0K-ThTqn6KsTjYkPjbLnW0krHbs0A4vTjYsQW0snj0snj0s0AdYTjYs0AwbUL0qn0KzpWYk0Aw-IWdsmsKhIjYs0ZKC5H00ULnqn0KBI1YknfK8IjYs0ZPl5fK9TdqGuAnqTZnVuyPJ0A-bm1dribGH0ZKCIZbq0Zw9ThI-IjYvndtsg1DdnsKYIgnqnHDdrHc3P10YnHD1rjfLPWRsnHm0ThNkIjYkPWfsn10sPjTdPW0d0ZPGujdbuWw-rAfsn10snj03m1w90AP1UHdaPjKjwW03nRD1nRR4nYcL0A7W5HD0TA3qn0KkUgfqn0KkUgnqn0KlIjYz0AdWgvuzUvYqn7tsg1Kxn7ts0Aw9UMNBuNqsUA78pyw15HKxn7tsg1Kxn0Ksmgwxuhk9u1Ys0AwWpyfqn0K-IA-b5iYk0A71TAPW5H00IgKGUhPW5H00Tydh5H00uhPdIjYs0A-1mvsqn0K9uAu_myTqnfK_uhnqn0KbmvPb5H0zwRfkrHDknYDYP1F7f1FafW0kPW9Anb7af16vnjfzwbRsPHckrDR3HdFiHb4af-w7ifKYTh7buHYvn10znjc0mhwGujYYnHfdnY77rH-7wDmLPRDLfRwKwHFjnWf3wH04wHNar0KBIjYs0Aq9IZTqn0KEIjYz0AqzTZfqninsc1DWPan4n1bzPj61P16WnansQW0snj0snansc1fWnanVc108nj0snj0sc1D8nj0snH0s0Z91IZRqPWRknjnsP0KkgLmqna3dnNtsQW0sg108njKxna3snNtsQW0zg108rHNxna3zn-ts0AF1gLKzUvwGujYs0ZFEpyu_myTqn0KWIWY0pgPxmLK95H00mL0qn0K-TLfqn0KWThnqnWbvPjb&us=newvui&xst=TjYkPjbLnW0krHbs0ynqnjF7wjD4nHD1fHfLnbNjnbFanjDvrDmzfRFjrjmsPjFAwH0dnWD3wH95R-FrHbFaNDNF0ycqPjDYPHPKwHb4wRwAP1NKPY7DfRRzf1cYrDRsrRRdfW6KT1YkPW04njbdn1RvnWmvPH6kPHDkP7tznWNxn07L5gw8uBd9UZKCmf7k5gw8uBvhsf7d5HmdnH01njfKIjYkPWfsn10sPjTd0ydk5H0an0cV0yPC5yuWgLKW0ykd5H0Kmv3qrjfsPHnsrH9xndqbusDkPjfdnjD1nHD4&word=&ck=1425.2.0.0.0.214.140.0&shh=www.baidu.com&sht=84053098_3_dg&wd=) | -0.3480 | 0.2227 | -0.0330 | 0.9107 |
| IL-6 | 0.0022 | 0.9941 | -0.2659 | 0.3581 |
| IL-10 | -0.0286 | 0.9227 | -0.3366 | 0.2392 |
| IL-2 | -0.1012 | 0.7306 | -0.2156 | 0.4591 |
